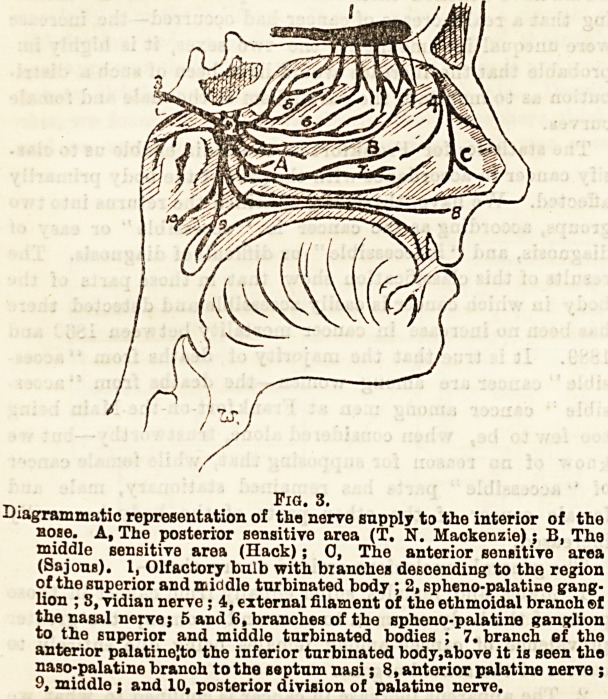# Rhinoscopy. II

**Published:** 1893-05-20

**Authors:** P. Watson Williams

**Affiliations:** Physician to the Throat Department Bristol Royal Infirmary


### RHINOSCOPY.—II.

P. Watson Williams, M.D.Lond., Physician to the Throat Department Bristol Royal Infirmary.

*The Physiology of the Nose* has received far less attention than the importance and various functions of the organ merits. Its functions are threefold—respiratory, olfactory, and vocal.

*The Function of the Nose in Respiration* is, perhaps, the most important and least appreciated of the three. The very complicated and peculiar formation of the passages, with an arrangement of the mucous membrane, provides a very large surface to which the inspired air is exposed in normal breathing, and by which means it is warmed and moistened before entering the bronchi. It can be readily conceived that the highly vascular mucous tissues covering the lower and middle turbinated bones and portions of the septum are capable of rapidly warming inspired air as it passes over them, and of secreting with equal rapidity a copious supply of watery mucus, which can yield its moisture to the warmed air. The functions of these bodies in nasal respiration has been so concisely, and in the main correctly described by Dr. Bosworth as long ago as 1886, that we quote this eminent authority *in extenso.* "The normal function of the mucous membrane is to secrete mucus, and only in such quantities as are sufficient to keep the membrane in a soft, moist, and pliable condition. Any excess of this amount becomes a morbid secretion. Normally nasal mucus is composed of 93 per cent. of water and 7 per cent. of solid matter. Robbed of a small portion of this water it becomes thick, inspissated, and unhealthy. Now, as we know, every breath of air that passes through the nasal chambers, and reaches the passages below, must become surcharged with moisture; otherwise it would rapidly exert a deleterious influence on the mucous membrane of the air passages beyond, by robbing them of their moisture, and so rendering their mucus thick and inspissated. It is estimated by physiologists that, in the course of twenty-four hours, about five thousand grains of water are taken up by the inspiratory current of air in its passage through the respiratory apparatus. Now, I think I am safe in saying that if five thousand grains of water were extracted from the mucous membrane of the bronchial tubes and air cells in the course of twenty-four hours, the result would be complete destruction of their function, to such an abnormally dry condition would they be reduced; for, as we know, in each act of inspiration the inspired air reaches only the larger bronchial tubes, and the source of moisture, therefore, of the inspiratory current cannot be from the smaller bronchial tubes or air cells. . . . Now, the mucous membrane of the lower air passages is endowed with no especial apparatus for the secretion of water; the only secretory apparatus with which it is endowed is in the mucous glands, which secrete mucus alone. In the nasal mucous membrane, however, we find an apparatus capable of furnishing this water, and this is the so-called erectile tissue of the turbinated bodies. This, then, is the great and prominent function of the nasal chambers to so prepare the ingoing current of air that it shall exercise no injurious influence on the mucous membranes of the passage below. . . . Now, unless the blood-vessels underlying a membrane called upon for this duty were very large and numerous, they would be inadequate to supply this large demand. Nature, therefore, has furnished the membrane in this region with such an abundant supply of large tortuous vessels that they assume the appearance of erectile tissue. . . . In addition to this large blood supply there must be, of course, some delicate mechanism by which their function is regulated. This control is exercised by the vaso-motor system of nerves. So delicately must this be arranged that the transudation of serum must accurately adapt itself to every existing atmospheric condition, . . . not only daily but even momentary changes in the humidity of the inspired air. It is easy to see, therefore, how great the demand must be upon the vaso-motor nerves which regulate the calibre of these blood-vessels, how constant the watchfulness which controls this endosmotic action, and therefore how easily any impairment of this function might occur."

The effects of the passage of inspired air through the nasal passages has been investigated and accurately determined by Aschenbrandt in 1886, and his results have been corroborated by Kayser, Block, and Greville Macdonald. The methods adopted by these observers differed only in detail, and consisted in making air, which had been inspired through the nose, pass into or through various tubes, where the changes in humidity and temperature could be noted. Greville Macdonald has further determined the chemical changes that take place in the atmosphere during its passage through the nose.

The conclusions arrived at by all these observers were very similar, and may be summarised as follows:

1. Whatever the temperature of the atmosphere, the air, after ordinary inspiration through the normal nasal passages, is always raised to the temperature of the blood before reaching the pharynx.

2. The air, after passing through the nose, was invariably saturated with moisture.

3. Greville Macdonald concludes that gaseous exchanges take place between the gases of the blood and those of the air, to a not inconsiderable extent. Moreover, the quantity of carbonic acid exhaled by the nasal mucous membrane is, in some measure, proportionate to the number of degrees in temperature to which the air is raised. This increase in the supply of heat is probably due partly to increased conduction, radiation, &c., of heat from the augmented blood supply to the mucous membrane, and partly by direct increase of oxidation in that and the subjacent structures.

The very abundant supply of racemose mucous glands in the naso-pharynx serve to aid the passage of food into the œsophagus.

FIG. 3.

Diagrammatic representation of the nerve supply to the interior of the nose. A, The posterior sensitive area (T. N. Mackenzie); B, The middle sensitive area (Hack); C, The anterior sensitive area (Sajous). 1, Olfactory bulb with branches descending to the region of the superior and middle turbinated body; 2, spheno-palatine ganglion; 3, vidian nerve; 4, external filament of the ethmoidal branch of the nasal nerve; 5 and 6, branches of the spheno-palatine ganglion to the superior and middle turbinated bodies; 7, branch of the anterior palatine to the inferior turbinated body, above it is seen the naso-palatine branch to the septum nasi; 8, anterior palatine nerve; 9, middle; and 10, posterior division of palatine nerve.

*The Nervous Functions of the Nose.*—As regards the function of smell little need be said here. I have already reminded the reader that the olfactory region occupies the upper portion of the nasal cavities as far down as the middle turbinated bone and the upper third of the septum. The peculiar character of the non-ciliated epithelium of this region and the nature of the terminations of the olfactory nerves are fully described in physiological text-books. We must, however, remember that for the function of smelling the mucous membrane must be in a moist condition, and free from accumulations of secretion, and also that loss of smell may be due to destruction of the olfactory bulbs or of the nerve fibres proceeding from it.

Of great importance is the consideration of the physiological reflexes of the nose, since, without a proper appreciation of their nature, it is difficult to understand the occurrence of many of the nasal neuroses. The physiological reflexes are sneezing, coughing, lacrymation, and vaso-motor changes giving rise to increased secretion. It is well known that bright sunlight often gives rise to sneezing, cough, lacrymation, and rhinorrhœa from excitation of branches of the fifth nerve. Dust and particles of foreign matter and irritating vapours on entering the nasal passages induce like phenomena. Three areas, termed the hyperæsthetic areas, have been determined, situated one on the posterior extremity of the inferior turbinated and the corresponding portion of the septum (J. N. Mackenzie), another at the anterior extremity of the inferior turbinated body (Hack), and the third in the mucous membrane of the vestibule (Sajous), excitation of which is particularly prone to give rise to nasal reflexes; and to these areas may be added the anterior extremity of the middle turbinated body to the corresponding portion of the septum, and the region of the Eustachian tubes. Dr. Greville Macdonald remarks that the cough which is excited by irritation of this area and of the posterior extremity of the inferior turbinated body is "hard, hollow, and, as the patient says, irritating, the sensation being referred to the neighbourhood of the larynx and trachea."

These sensitive areas correspond with the ramifications of the ethmoidal branch of the nasal nerve and branches from Meckel's ganglion, which are the sensory nerves of the nasal fossæ.

## Figures and Tables

**Fig. 3. f1:**